# Analyzing the role of customers’ experiences and emotional responses in shaping Generation Z’s impulse buying behavior on Shopee video platform

**DOI:** 10.1371/journal.pone.0322866

**Published:** 2025-05-05

**Authors:** Thi Thuy An Ngo, Hoang Lan Thanh Nguyen, Ho Truc Anh Mai, Hoang Phi Nguyen, Thi Huyen Tran Mai, Phuoc Long Hoang

**Affiliations:** 1 Department of Soft Skills, FPT University, Can Tho, Vietnam; 2 Department of Business Administration, FPT University, Can Tho, Vietnam; Wenzhou-Kean University, CHINA

## Abstract

The swift growth of e-commerce has markedly changed how consumers shop, especially among Generation Z, which is called Digital Natives. This study examines how product presentation videos on the Shopee video platform influence impulse buying behaviors in this group, focusing on how internal stimuli, including entertainment experience (ET), educational experience (ED), escapist experience (ES), and esthetic experience (EH) influence online impulse buying (OIB) through the mediation of arousal (AR) and pleasure (PL). In addition, demographic factors, including age, gender, and income, are treated as control variables. This research adopts a quantitative methodology, and data was gathered using a Likert scale questionnaire and a non-probability sampling method, while the SmartPLS statistical tool was used to analyze the interactions of these stimuli and their effect on the impulse buying behavior of Generation Z on digital platforms. Research indicates that entertainment and recreational activities boost emotional engagement by eliciting arousal and pleasure. Educational experiences increase knowledge and also stimulate these feelings. Escapist activities provide temporary relief from daily stresses, increasing arousal, but can also highlight personal insecurities, possibly reducing pleasure. Esthetic experiences, subject to personal tastes, provoke emotional reactions that may vary in pleasure. For Generation Z, arousal and pleasure significantly influence impulsive buying decisions. The insights indicate that effectively managing internal factors can trigger emotions leading to impulsive purchases, offering strategic marketing tactics for optimizing e-commerce on platforms like Shopee video. This research advances the understanding of consumer behavior theories in the digital era, emphasizing the intricate roles of arousal and pleasure in online impulse buying.

## 1. Introduction

E-commerce has significantly transformed consumer shopping habits, experiencing rapid growth over the past decade. This transformation is primarily driven by the widespread availability of the internet and the increasing use of smartphones, which have become essential tools for online shopping. Globally, e-commerce sales are projected to exceed $5 trillion by 2025, highlighting the sector’s rapid expansion [1]. Southeast Asia, in particular, is emerging as a critical player in the global e-commerce landscape. With over 400 million internet users, representing 70% of the region’s population, Southeast Asia is witnessing remarkable growth in e-commerce activities [2]. The region’s gross merchandise value (GMV) is expected to reach $330 billion by 2025, driven by a compound annual growth rate (CAGR) of 22% [1]. This growth is propelled by widespread internet access and a mobile-first approach, with 72.6% of users accessing the internet via smartphones [3].

A significant aspect of this digital expansion is the rise of short-form video shopping platforms, such as Shopee video, which are reshaping traditional consumer behaviors by providing personalized and interactive shopping experiences. This shift is particularly pronounced among Generation Z, individuals born between 1997 and 2012 [4], who are driving a fundamental change in consumer engagement within digital spaces [5]. Platform like Shopee and TikTok Shop dominate the online retail market in Southeast Asia, accounting for over 90% of the sector, with Shopee alone holding 71.4% of the market share in Quarter 2, 2024 [6] Vietnam, with one of the highest internet penetration rates in Southeast Asia at 80%, plays a pivotal role in this regional surge [2]. The country’s GDP is projected to reach $369.4 billion by 2024, driven by robust economic activities and digital advancements [7].

The emergence of short-form video shopping platforms represents a transformative shift in how consumers engage with e-commerce. Short-form videos, typically lasting under 60 seconds [8], have proven highly effective in driving impulse buying behavior by delivering focused, engaging content that quickly captures consumer attention [9]. These videos are designed to utilize emotional triggers, create a sense of urgency, and include clear calls to action, prompting rapid purchasing decisions [10]. This significant shift towards short-form content is a response to changing consumer behaviors, where diminishing attention spans and an overwhelming volume of available content have led to a preference for quick, easily digestible videos that convey essential messages in just a few seconds [11]. The shift is further reinforced by the evolution of social media platforms, which prioritize and promote short-form videos through their algorithms, leading to higher engagement rates [12]. These videos often go viral, significantly boosting brand visibility and impact. As a result, businesses are compelled to rethink their marketing strategies, focusing on creating immediate, emotionally resonant, and visually appealing content that captures attention and drives conversions in an increasingly competitive digital landscape. In this rapidly evolving environment, understanding and leveraging the power of short-form video content is crucial for businesses to stay competitive and effectively engage their target audience.

Previous research has underscored the significance of website design and emotional factors in shaping consumer shopping behavior [13,14].These studies have primarily focused on optimizing online retail experiences through website enhancements, aiming to improve user engagement and satisfaction. However, a critical gap remains in understanding how short-form video content specifically impacts online impulse buying behavior. Numerous of the existing literature has focused on physical store environments or traditional web interfaces, which do not fully capture the unique dynamics of short-form videos. For instance, studies by Sands et al. [15] and Kastenholz et al. [16] explored emotional responses such as pleasure and arousal, with Sands et al. [15] focusing on event themes in retail settings and Kastenholz et al. [16] examining emotional experiences through face-to-face interactions. While these studies provide valuable insights into emotional drivers, they fall short of addressing the specific characteristics and effects of short-form video content. Similarly, Cheng et al. [17] investigated the emotional triggers of impulse buying but did not delve deeply into the role of short-form videos in this context. Moreover, Ngo et al. [10] highlighted the impact of external factors on emotions that influence impulse buying but did not account for the role of internal factors, such as the consumer’s intrinsic motivations and psychological states, which can be significantly affected by short-form video content. Linden et al. [18] further recommended that future research should differentiate between positive and negative arousal, particularly in the context of short-form video content, to better understand how these emotional states influence consumer behavior.

This study aims to bridge this gap by exploring how emotional factors, such as arousal and pleasure triggered by product demonstration videos, influence Generation Z’s impulse buying behavior on the Shopee video platform. By focusing on internal factors such as entertainment, education, escape, and esthetic experience, this study provides valuable insights into the emotional drivers of impulse buying in digital environments. These insights are crucial for marketers and e-commerce platforms seeking to optimize their strategies to enhance consumer engagement. The findings of this study will enable marketers to create engaging video content that resonates with Gen Z’s preferences, thereby improving the effectiveness of marketing strategies on platforms like Shopee. Moreover, the study offers practical recommendations for enhancing the design and functionality of e-commerce platforms to stimulate impulse purchases, ultimately enriching the overall online shopping experience.

## 2. Literature review

### 2.1. Online impulse buying (OIB)

Impulse buying is characterized by an overwhelming and spontaneous urge to purchase items, often driven by immediate emotional reactions rather than deliberate decision-making processes [19,20]. This behavior can disrupt a consumer’s psychological equilibrium, leading to feelings of post-purchase guilt or regret [21,22]. Common indicators of online impulse buying include frequent spontaneous purchases, an inability to resist buying urges, and subsequent negative emotional responses [23].

Technological advancements in digital shopping have significantly transformed the retail landscape, simplifying the purchasing process and making impulse buying more prevalent than in traditional shopping environments [24,25]. This behavior becomes especially pronounced in online settings, where a variety of factors can trigger impulsive purchases [21]. The convenience of online shopping, coupled with the allure of flash sales and time-limited offers, can significantly increase the likelihood of impulsive buying, particularly when consumers are emotionally vulnerable [26–28]. The design and functionality of online shopping platforms play a crucial role in enhancing impulse buying behavior. Elements such as visual appeal, intuitive navigation, one-click purchasing options, and social proof mechanisms (e.g., customer reviews and ratings) contribute to a seamless shopping experience that encourages impulsivity [29]. Moreover, personalized recommendations and streamlined purchasing processes further facilitate immediate purchases, making it easier for consumers to act on their impulses and driving higher conversion rates for online retailers [30,31]. Research has shown that the perceived quality of an offer plays a crucial role in building trust [32] and is considered one of the primary factors influencing trust [33]. Within product recommendations, there is an expectation that as consumers view algorithms as effective and capable of delivering high-quality suggestions, their trust in these recommendations increases [34]. This trust serves to deepen the consumer’s experience, subsequently elevating the propensity for impulse purchases.

### 2.2. The experience economy framework (4Es)

The Experience Economy framework (4Es), conceptualized by Pine and Gilmore [35], delineates the transition from service-based economies to experience-based economies, highlighting the significance of creating memorable and engaging consumer experiences. This framework categorizes experiences into four distinct types: entertainment, educational, escapism, and esthetic, collectively referred to as the 4Es. The “sweet spot” is achieved when these elements are harmoniously combined, providing the richest consumer experiences [35].These experiences vary in consumer engagement levels, ranging from passive to active, and the nature of the consumer’s connection to the experience. According to Sundbo and Sorensen [36], an experience occurs in the mind, influenced by external stimuli and shaped by individual motivations, needs, and personal strategies. They argue that experiences can be triggered by stimuli affecting all senses, thus enhancing traditional goods and services and playing a crucial role in contemporary marketing [37].

The 4Es framework is particularly relevant for research in digital marketing and consumer engagement, as it provides a versatile tool for analyzing various types of consumer interactions [38,39]. It underscores the importance of consumer engagement, a critical factor in the digital age where capturing and retaining consumer attention is increasingly challenging [40]. The framework is well-suited for analyzing digital platforms like Shopee video, which use multimedia capabilities to engage consumers through rich, interactive experiences. The framework helps in identifying which of the 4Es, entertainment, educational, escapism, and esthetic, which are most effective in engaging consumers on the Shopee video platform. It also facilitates the measurement of consumer engagement levels (passive vs. active) and assesses how these experiences influence overall consumer arousal and pleasure, potentially leading to impulse buying.

In addition to the 4Es framework, other theories such as Flow Theory, proposed by Csikszentmihalyi [41], explore the emotional aspects of consumer experiences. Flow Theory examines the state of being fully immersed and engaged in an activity, a concept frequently applied to user experiences in digital environments [42]. While Flow Theory focuses on specific emotional states and engagement levels, the 4Es framework offers a more comprehensive approach by integrating cognitive and emotional aspects across a broader spectrum of experiences. This comprehensive nature makes the 4Es framework particularly suitable for analyzing the complex and multifaceted nature of consumer interactions in the digital age.

### 2.3. Entertainment experience (ET)

The strategic use of product presentation videos on the Shopee video platform leverages entertainment experiences to enhance customer arousal and pleasure, which are key drivers of online impulse buying. These experiences, characterized by fun, amusement, and surprise, create a positive and enjoyable shopping environment [35]. Entertainment experiences are also considered a critical factor in creating the ‘wow’ effect, which refers to customers’ reactions of astonishment and strong impressions [43]. Such reactions can lead customers to share their experiences on social media, indicating that entertainment factors are closely associated with customer satisfaction, loyalty, and willingness to recommend [44]. For instance, integrating humor into product descriptions, video commercials, or interactive features can entertain customers and foster a positive shopping experience [45]. The type of entertainment provided is often tailored to the target audience and brand image. For example, platforms targeting young adults may employ interactive quizzes and gamification, while luxury brands may opt for high-quality video testimonials with subtle animations [46].

Entertainment experiences have a notable influence on Generation Z, as they shape perceptions and behaviors toward online shopping by offering a fun, engaging, and imaginative experience [47]. This influence extends to their attitudes, intentions, and actions as consumers [25]. Research indicates that engaging and emotionally resonant video content can generate high levels of arousal and pleasure, leading to impulse buying behavior [48]. A study by Liu et al. [49] suggests that entertainment experiences, particularly in physical retail environments, create an immersive setting where consumers feel more relaxed and detached from financial concerns. This relaxation often lowers the mental barriers that typically govern planned purchasing decisions, thereby promoting impulsive shopping behavior. Furthermore, the entertainment factor can distract consumers from their initial shopping plans, redirecting their attention to new products or services, which may stimulate unplanned purchasing behaviors [49,50]. The sensory-rich environment of videos, including visual and auditory stimuli, captures attention and evokes emotional responses that can override rational decision-making processes, resulting in impulsive purchases.

Yi and Jai [51] suggest that entertainment value correlates with individuals’ emotions and psychosocial motivations, creating a user interface that enhances positive consumer emotions online. According to Rajan [52], businesses leverage the entertainment experience in sales videos to capture consumer interest, with entertaining videos increasing the likelihood of impulsive purchases. This is attributed to the activation of the brain’s reward system, which releases dopamine, enhancing feelings of satisfaction and reducing self-control. Therefore, consumers may make unplanned purchases to prolong or re-experience the pleasurable feeling [52,53]. Based on these insights, the following hypotheses were proposed:

**H1:** Entertainment experience has a significant positive impact on arousal.

**H2:** Entertainment experience has a significant positive impact on pleasure.

### 2.4. Educational experience (ED)

The concept of educational experience, as introduced by Pine and Gilmore [35], goes beyond mere information provision by actively engaging customers in learning processes that foster knowledge acquisition and skill development. This engagement enhances customer experience and pleasure across various sectors. With the rise of digital technology, platforms such as mobile apps, interactive kiosks, and online media have become effective channels for delivering educational content, providing convenience and accessibility [54].

Educational experiences enable customers to use products and services correctly and effectively, allowing them to fully realize the value of these offerings and better meet their needs [55]. Peng and Li [56] argue that these experiences not only impart information and knowledge but also help consumers perceive deeper value through interactive activities. When consumers feel educated and informed about a product, they tend to experience higher levels of arousal and increased pleasure, which can lead to impulsive purchase decisions without extensive deliberation. Instructional or product introduction videos are a prime example of online educational experiences. According to Xu and Pratt [57], these videos can create a sense of excitement and stimulate customers’ curiosity, thereby promoting impulsive purchasing decisions. Learning experiences can enhance the way customers perceive the product’s value, increasing their willingness to accept risks and make quick purchase decisions without lengthy deliberation. When customers feel they thoroughly understand the product, they are more inclined to make impulsive decisions, believing their choice to be ‘smart’ and considered, although emotional factors still play a significant role [58]. This is especially evident in unboxing and product review videos on platforms like Shopee video platform, where consumers are drawn to realistic experiences and can easily imagine owning the product.

The provision of valuable information in product introduction videos conveys the company’s goodwill and commitment to customer satisfaction, encouraging product or service usage and feedback sharing. During this process, customers are stimulated, their overall experience is enhanced, and their pleasure increases. Jai and Yi [51] found that factors such as informational content, presentation style, and interactivity significantly impact consumer emotions. They observed that well-designed educational experiences, which creatively and interactively deliver knowledge, can lead to increased impulsive purchases as customers feel immediately stimulated and satisfied.

Moreover, customizing educational content to align with individual preferences and interests maximizes its relevance and effectiveness, underscoring the importance of personalization in educational experiences [59]. The success of educational initiatives depends on tailoring them to the specific industry context and the target audience’s preferences. For Generation Z, education is perceived as a lifelong pursuit [60]. Therefore, the pleasant arousal derived from educational experiences can empower Generation Z customers and increase their confidence in purchasing decisions [38,61]. Based on the literature review, the following hypotheses were proposed:

**H3:** Educational experience has a significant positive impact on arousal.

**H4:** Educational experience has a significant positive impact on pleasure.

### 2.5. Escapist experience (ES)

The concept of escapist experience, introduced by Pine and Gilmore [35], involves immersing customers in a world distinct from their everyday lives, providing pleasure and arousal that can lead to impulsive purchases [62]. Technological advancements, such as more engaging video games, continuous access to social networks, and enhanced device portability, have facilitated the desire for escapism, particularly among Generation Z [63]. This generation, profoundly shaped by digital technology [64], has a deep connection with the internet, social media, and digital entertainment, making immersive digital experiences a natural form of escape [65].

Generation Z faces significant stress and anxiety due to various factors, including academic pressures, social media influence, economic uncertainties, and global issues like climate change [66]. These challenges prompt them to seek temporary relief through escapist activities, often influenced by a media and cultural environment that celebrates escapism [67]. They find enjoyment, relief, and connection in immersive digital environments, which foster emotional engagement and encourage impulsive purchases as a means of connecting with the brand’s escapist narrative. According to Tumbat and Belk [68], escapist scenarios stimulate customers to step out of their normal state, making them more susceptible to impulsive shopping motivations. In digital environments, particularly on video platforms like YouTube, TikTok, and Shopee, viewers can become engrossed in scenes of travel, luxury experiences, or highly engaging entertainment, prompting them to purchase related products or services to experience a similar sense of escape [69]. When customers are exposed to videos showcasing an appealing escapist setting (such as travel destinations, luxury hotels, or tech products), they are likely to enter a mindset of desiring that experience for themselves, leading to unplanned purchases [70–72]. The visual and audio elements in the videos enable customers to perceive the escapist environment more vividly and realistically, thus stimulating impulsive buying behavior.

Many consumers, including Generation Z, shop for pleasure and leisure, not merely to acquire items. Enhancing the escapist aspects of the shopping environment adds significant value [73]. Experiences provided by product introduction videos offer a novel and immersive escape, increasing arousal and leading to the discovery of new products. The novelty and immersive qualities of these experiences can prompt impulse buying [50]. Based on these insights, the following hypotheses were proposed:

**H5:** Escapist experience has a significant positive impact on arousal.

**H6:** Escapist experience has a significant positive impact on pleasure.

### 2.6. Esthetic experience (ET)

Pine and Gilmore [35] emphasized the significance of esthetic experiences, which involve the appreciation of beauty and sensory appeal, particularly in e-commerce platforms that utilize well-designed interfaces. Generation Z, raised in a digital era dominated by visually-driven platforms, places high value on aesthetically pleasing products and environments [74]. As a result, esthetic experiences play a crucial role in shaping this generation’s consumer behavior by leveraging their strong preference for visual and sensory appeal.

Platforms like Shopee’s video likely capitalize on esthetic experiences to influence impulse buying behaviors among Generation Z by showcasing products through visually engaging content and multiple perspectives. Research suggests that effective visual merchandising, including thoughtful product selection, color schemes, and display arrangements, significantly affects online consumer behavior [75]. Huang [76] and Zhang et al. [12] found that platforms featuring visually appealing layouts and high-quality images enhance customer pleasure and perceived value. Similarly, Ryu and Ryu [77] noted that the visual appeal of products in online shopping environments evokes feelings of arousal and fluency, which profoundly influence consumer behavior.

Kumar and Kim [72] found that esthetically rich videos, with elements like vibrant colors, lighting, and music, evoke arousal and pleasure, often prompting impulsive purchases. In fashion and cosmetics, in particular, esthetic videos inspire a desire for ownership, allowing customers to envision the luxury and style conveyed in the visuals [78]. Similarly, Verhagen and Van Dolen [29] suggest that a visually appealing environment and strong aesthetic attraction create a “must-have” feeling, motivating immediate purchase. Videos designed with captivating imagery and lighting not only evoke a sense of luxury but also ignite the desire to attain a similar experience. Beautifully crafted videos not only evoke a sense of luxury but also stir the desire to experience the “ideal lifestyle” depicted in the video, encouraging customers to make purchases to achieve their aspirational self-image.

Moreover, Lee et al. [59] explored impulse buying behavior on online apparel websites, demonstrating that visual appeal and sensory-rich environments strongly drive Generation Z’s purchasing decisions. According to Hsieh et al. [79], high levels of arousal and pleasure, prompted by esthetic design and effective marketing strategies, can significantly enhance impulse buying behaviors. Based on these findings, the following hypotheses were proposed:

**H7:** Esthetic experience has a significant positive impact on arousal.

**H8:** Esthetic experience has a significant positive impact on pleasure.

### 2.7. Arousal (AR) and Pleasure (PL)

Arousal, defined as the level of sensory stimulation, energy, or excitement, plays a critical role in shaping consumer behavior [80,81]. The relationship between arousal and consumer responses is complex. Research suggests that moderate levels of arousal can lead to pleasurable experiences, whereas excessive arousal may result in negative responses [81,82]. Pleasure, which encompasses feelings of satisfaction and happiness, is closely linked to arousal. This emotional state can vary from high-arousal emotions like joy to low-arousal states such as relief and contentment [80,83].

The Pleasure, Arousal, Dominance (PAD) model proposed by Mehrabian and Russell [84] suggests that emotions significantly influence personal reactions and environmental perceptions. Within this framework, arousal and pleasure are key dimensions that interact to influence consumer responses [83]. Numerous studies, both in traditional and technology-based retail contexts, have demonstrated a positive and direct relationship between arousal and pleasure [85,86]. Without arousal, individuals may not experience pleasurable emotions in response to stimulating cues [87]. In the context of online shopping, arousal has been found to have a direct correlation with pleasure, both of which significantly impact impulse purchasing behavior [88,89]. For instance, Kim and Johns [89] found that positive emotions such as arousal and pleasure, experienced during online shopping, increase the likelihood of consumers making impulsive purchases. This suggests that the emotional responses elicited by the shopping experience play a crucial role in influencing purchasing decisions.

Moreover, pleasure and arousal induced by various online visual merchandising cues can greatly affect consumer satisfaction, purchase intention, and approach behavior [90]. Mattila and Wirtz [91] suggested that heightened arousal can weaken self-control, as positive emotions often lead individuals to process information more intuitively [92], and to be more inclined toward self-reward [93]. High levels of both arousal and pleasure are associated with enhanced positive feelings toward purchasing, making consumers more inclined to engage in unplanned purchases driven by the desire for immediate gratification [94,95]. This demonstrates that the emotional environment created by online retailers can significantly influence consumer behavior, particularly in encouraging impulse buying. Based on these insights, the following hypotheses were proposed:

**H9:** Arousal has a significant positive impact on pleasure.

**H10:** Arousal has a significant positive impact on online impulse buying.

**H11:** Pleasure has a significant positive impact on online impulse buying.

### 2.8. Demographic factors

Demographic characteristics play a crucial role in influencing consumer behavior, particularly in the context of impulse buying. Key factors such as age, gender, and income level have been identified as significant determinants of impulsive purchasing tendencies [96]. These factors contribute to variations in consumer behavior across different segments of the population, with each demographic group exhibiting distinct traits, preferences, and responses to marketing stimuli.

Age is one of the most influential demographic factors affecting impulse buying behavior. Research shows that consumers across various age groups exhibit unique purchasing patterns, shaped by their technological proficiency, lifestyle, and exposure to online marketing. Impulse buying behavior has been found to have a significant inverse relationship with age; as consumers advance in age, their propensity for impulsive purchasing diminishes [97]. Younger consumers, for instance, tend to have higher technological adeptness and are more frequently exposed to digital marketing strategies, which increases their susceptibility to impulsive buying [98]. In contrast, older consumers may demonstrate different purchasing behaviors due to their varying levels of technological engagement and spending priorities [99,100].

Gender also plays a significant role in shaping impulse buying behavior. Studies indicate that women are more prone to impulsive purchases based on emotional factors [101,102], especially in categories such as fashion and beauty products [103]. In contrast, men tend to make purchases based on prior planning and budgeting [104]. This tendency is often driven by emotional factors, including the desire for immediate satisfaction, self-expression, or mood enhancement [105].

Income level is another critical determinant of impulse buying behavior. Tariq Jalees et al. [106] demonstrated that income has a significant impact on consumers’ impulsive behavior, with higher income levels correlating with a greater tendency for unplanned purchases. Consumers with more financial resources are generally more willing and able to indulge in spontaneous purchases, as the financial impact of such decisions is less significant for them [107]. Moreover, disposable income can act as a constraint on impulse purchases, fostering apprehension and contributing to consumer resistance [108]. Based on these findings, the following hypotheses were proposed:

**H12a:** Age significantly controls the likelihood of engaging in online impulse buying behavior.

**H12b:** Gender significantly controls the likelihood of engaging in online impulse buying behavior.

**H12c:** Income significantly controls the likelihood of engaging in online impulse buying behavior.

### 2.9. Theoretical framework

**Fig. 1:**
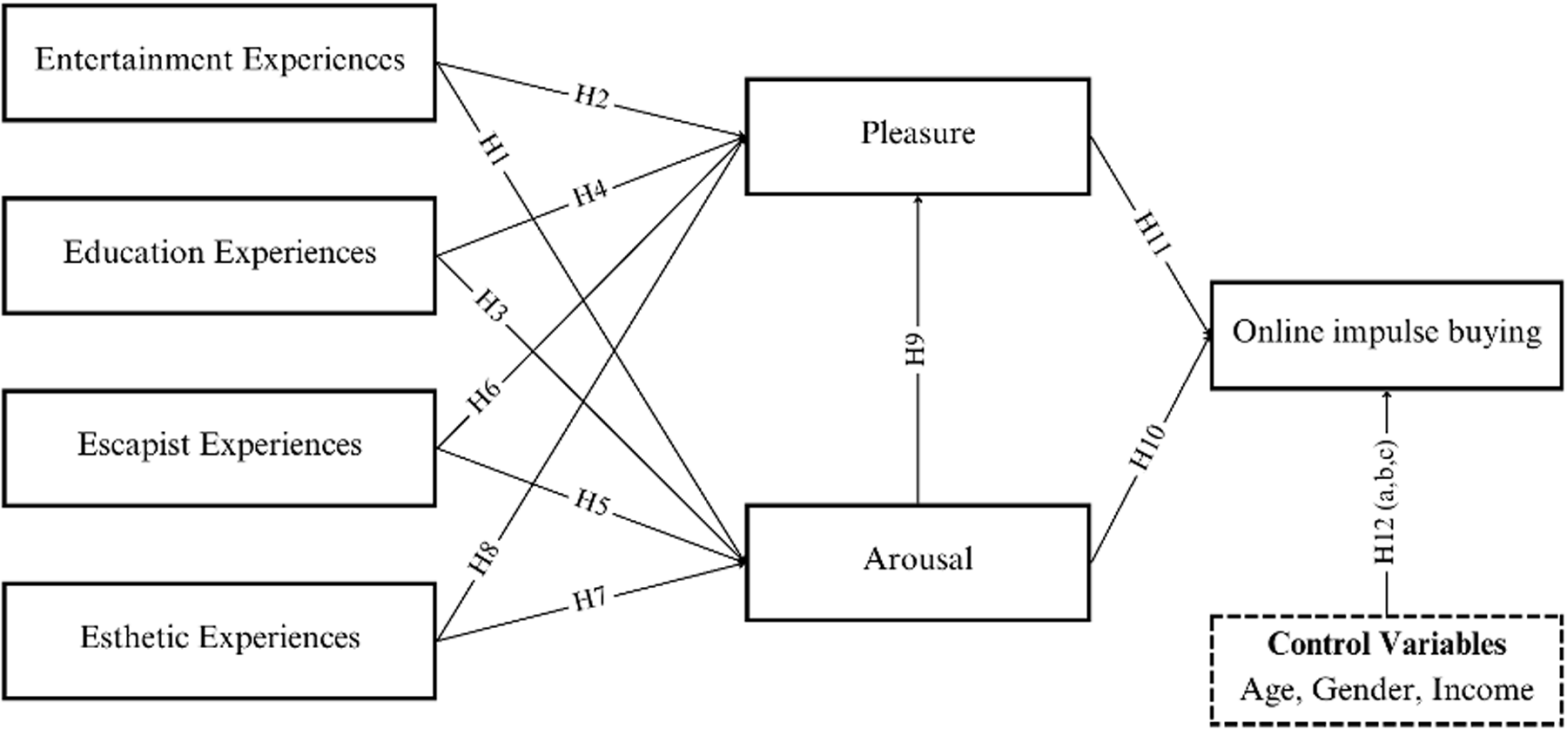
Theoretical framework.

This study aims to thoroughly investigate the internal factors that influence online impulse buying on Shopee’s video platforms. These factors, rooted in an economic framework, encompass the entertainment, educational, escapist, and esthetic experiences. The research also utilizes the PAD emotional state model, which evaluates emotional responses based on pleasure and arousal, to gain insights into the emotional triggers behind online impulse buying. Additionally, demographic factors such as age, gender, and income are considered to understand their direct control on online impulse buying behavior. The proposed research framework is demonstrated in Fig 1.

## 3. Method

### 3.1. Participants

This study employed a quantitative research methodology to investigate the factors influencing the impulse buying behaviors of Vietnamese Generation Z consumers on the Shopee video platform. The sample consisted of 438 participants born between 1995 and 2006, thereby excluding minors. Participants were selected based on their experience with spontaneous purchases on Shopee, and informed written consent was obtained from all participants.

The demographic composition of the sample included 150 males (34,2%), 288 females (65,8%). The sample predominantly consisted of young, educated individuals with varying monthly incomes: 180 participants (41.1%) earned less than 5 million VND, 114 participants (26%) earned between 5 and 10 million VND, 84 participants (19.2%) earned between 10 and 15 million VND, 38 participants (8.7%) earned between 15 and 20 million VND, and 22 participants (5%) earned more than 20 million VND. Variations were observed in impulsive purchase frequency and amounts of impulsive purchases. Specifically, 289 participants (66%) reported making impulsive purchases fewer than three times per month, 96 participants (21.9%) reported making three to five impulsive purchases per month, and 53 participants (12.1%) reported making more than five impulsive purchases per month. Regarding the types of products purchased, 180 participants (41.1%) reported buying cosmetics. This was followed by electronic equipment, with 66 participants (15.1%), and household appliances, with 58 participants (13.2%). Fashion items were purchased by 34 participants (7.8%). Additionally, 100 participants (22.8%) indicated that they bought other types of products not specifically categorized in the provided options (see Table 1).

**Table 1:** Demographic information and purchase behavior of respondents.

Categories	Number of Respondents	Percentage
**Gender**		
Male	150	34.2
Female	288	65.8
**Age**		
From 19972001	149	34
From 20022006	289	66
**Income**		
Under 5 million VND	180	41.1
From 5 - under 10 million VND	114	26
From 10 - under 15 million VND	84	19.2
From 15 - under 20 million VND	38	8.7
Above 20 million VND	22	5
**Purchase Frequency on the Shopee video Platform**		
Less than 3 times	289	66
From 3 to 5 times	96	21.9
More than 5 times	53	12.1
**Items Purchased on the Shopee video Platform**		
Cosmetics	180	41.1
Household appliances	58	13.2
Electronic equipment	66	15.1
Fashion items (clothes, shoes, accessories, etc.)	34	7.8
Other	100	22.8

### 3.2. Instruments

This study utilized a structured questionnaire as the primary instrument, which was divided into three sections. The first section collected demographic information, including age, gender, educational level, occupation and income. The second section focused on purchasing behavior on Shopee, such as the frequency of spontaneous purchases. The third section measured the study’s variables using a five-point Likert scale, ranging from strong disagreement (1) to strong agreement (5).

The questionnaire was adapted from validated scales and measurement tools from previous research, including Huang et al. [109], Hsieh et al. [110], Kim et al. [89], Mehrabian and Russell [84], and Rook and Fisher [111]. The instrument was reviewed by a marketing research expert to ensure its validity. The questionnaire covered seven variables, including Entertainment experience, Educational experience, Escapist experience, Esthetic experience, Arousal, Pleasure, and Online impulse buying, which were assessed through 28 items as presented in Table 2.

**Table 2:** Measurement model assessment.

Construct	Items	Outer loadings	CA	CR	AVE
Entertainment experience [38]	ET1: “The way products were presented on Shopee video was amusing to me.”	0.892	0.900	0.931	0.770
	ET2: “The way products were presented on Shopee video was very entertaining.”	0.897			
	ET3: “I really enjoyed looking at the new product presentations on Shopee video.”	0.865			
	ET4: “I feel that it is pleasant to use Shopee video for shopping.”	0.856			
Educational experience [38]	ED1: “The product presentations created a shopping experience that was educational to me.”	0.888	0.893	0.926	0.757
	ED2: “Browsing product presentations on Shopee video stimulated my curiosity to learn new things about products.”	0.872			
	ED3: “The product presentations on Shopee video have made me more knowledgeable about products.”	0.857			
	ED4: “I learned about products while browsing the product presentations on Shopee video.”	0.863			
Escapist experience [38]	ES1: “When looking at the product presentations on Shopee video, I felt I was in a different world.”	0.882	0.918	0.942	0.802
	ES2: “I felt like I was a different person while looking at the product presentations on Shopee video.”	0.909			
	ES3: “I totally forgot about my daily routine while looking at the product presentations on Shopee video.”	0.903			
	ES4: “While looking at the product presentations on Shopee video, I completely escaped from reality.”	0.890			
Esthetic experience [38]	EH1: “The product presentations of Shopee video provided pleasure to my senses.”	0.889	0.909	0.936	0.785
	EH2: “Shopee video’s product presentations were very attractive.”	0.887			
	EH3: “Shopee video product presentations really showed attention to design detail.”	0.875			
	EH4: “Just looking at the product presentations on Shopee video was very pleasant.”	0.892			
Arousal [109,110]	AR1: “When I was shopping on Shopee video, I felt excited.”	0.924	0.928	0.949	0.823
	AR2: “When I was shopping on Shopee video, I felt active.”	0.902			
	AR3: “When I was shopping on Shopee video, I felt aroused.”	0.904			
	AR4: “When I was shopping on Shopee video, I felt stimulated.”	0.899			
Pleasure [89,109,110]	PL1: “When I was shopping on Shopee video, I felt happy.”	0.879	0.915	0.94	0.798
	PL2: “When I was shopping on Shopee video, I felt pleased.”	0.913			
	PL3: “When I was shopping on Shopee video, I felt satisfied.”	0.880			
	PL4: “When I was shopping on Shopee video, I felt hopeful.”	0.900			
Online Impulse Buying [84,111]	OIB1: “I usually buy products on Shopee videos spontaneously.”	0.895	0.905	0.933	0.778
	OIB2: “The products I bought on Shopee video are mostly unplanned.”	0.902			
	OIB3: “I bought products on Shopee video that I did not initially want to buy.”	0.865			
	OIB4: “I sometimes cannot suppress the feeling of wanting to buy something online.”	0.865			

### 3.3. Data collection

This study utilized a convenience sampling method with a structured questionnaire to collect data. This method was chosen for its efficiency and cost-effectiveness, allowing access to participants within a limited time [112]. The survey questionnaire was distributed widely and randomly, both online and offline to ensure a diverse participant sample. Online surveys were conducted using Google Forms and distributed via email and social media platforms, including Facebook, Instagram, and Zalo. Concurrently, physical questionnaires were distributed in public places such as workplaces and university campuses.

The data collection was carried out from 5^th^ April to 27^th^ April 2024 and received over 600 responses. After filtering out responses that exhibited criteria such as uniformity, inconsistency, incomplete data, or overly quick completion times of under two minutes, 438 valid responses were retained. This sample size met the requirements for thorough analysis. According to Hair et al. [113] Partial Least Squares Structural Equation Modeling (PLS-SEM) requires a minimum sample size of at least 10 times the number of predictor variables in the model. Given that the study involved 28 predictor variables, a minimum sample size of 280 participants was necessary to ensure statistical robustness.

Ethical considerations were a priority. The survey introduction outlined the study’s objectives and assured participants of confidentiality and data protection. Written consent was obtained through a check box at the start of the survey, allowing respondents to confirm their voluntary participation and authorize the use of their data for research purposes. The study received ethical approval from the Board of Directors at FPT Can Tho University, Vietnam (Approval No. 20240402.06), adhering to all standards for research involving human participants.

### 3.4. Data analysis

Data were analyzed using Partial Least Squares Structural Equation Modeling (PLS-SEM) with SmartPLS 4.0 software. PLS-SEM was chosen for its flexibility and effectiveness in achieving prediction accuracy, even with smaller sample sizes and non-normal data distributions [114]. The analysis process involved a two-step approach [115]. The first step is the assessment of the measurement models. This step employs various measures to assess the quality and reliability of the dataset, including Cronbach’s alpha and composite reliability for evaluating internal consistency, as well as convergent validity and discriminant validity to ensure the validity of the constructs [116,117]. The second step is the assessment of the structural model. This includes evaluating indices such as collinearity, path coefficients, explanatory power, and predictive power. Bootstrap analysis was conducted to rigorously test the study framework and hypotheses, facilitating the examination of relationships among latent variables, exploring correlations, and establishing statistical significance. This comprehensive data analysis approach ensured the reliability and validity of the study’s findings.

## 4. Results

### 4.1. Measurement model results

#### 4.1.1. Indicator reliability, convergent validity and reliability of constructs.

The measurement model was meticulously evaluated to ensure the validity and reliability of the collected data. This evaluation process focused on three critical aspects: indicator reliability, convergent validity, and construct reliability. The analysis utilized outer loading coefficients, Cronbach’s alpha (CA), composite reliability (CR), and average variance extracted (AVE) as key metrics.

Indicator reliability assesses how consistently individual indicators (observed variables) reflect the underlying construct they are designed to measure. According to Hair et al. [115], an outer loading coefficient above 0.7 is recommended as an indicator of reliability. As detailed in Table 2, the outer loading coefficients for all observed variables range from 0.856 to 0.924, all of which exceed the recommended threshold, confirming the high reliability of the indicators used in this study.

Construct reliability evaluates the internal consistency of the indicators within each construct, ensuring that they collectively measure the same underlying concept. This is assessed using Cronbach’s alpha (CA) and Composite Reliability (CR) indices, both of which should be greater than 0.7 to confirm reliability [115]. As presented in Table 2, the Cronbach’s alpha values for all constructs range from 0.900 to 0.928, while the CR indices range from 0.926 to 0.949, all above the 0.7 threshold. These results strongly support the reliability of the constructs.

Convergent validity determines whether the indicators of a construct are indeed related, meaning they converge to measure the same underlying concept. This is measured using the Average Variance Extracted (AVE), where a value of 0.5 or higher indicates sufficient convergent validity [115]. The AVE values for all constructs, which range from 0.757 to 0.823 as shown in Table 2, confirm that the constructs have adequate convergent validity.

Overall, the measurement model successfully meets all essential criteria for indicator reliability, convergent validity, and construct reliability. As evidenced by the results in Table 2, all indicator values for each construct surpass the established thresholds, thereby ensuring the robustness of the analysis and reinforcing the validity and reliability of the data.

#### 4.1.2. Discriminant validity of constructs.

Discriminant validity is a fundamental aspect of construct evaluation, ensuring that a given construct is distinct from others within a theoretical model. Two widely accepted methods for assessing discriminant validity are the Fornell-Larcker criterion and the Heterotrait-Monotrait Ratio of Correlations (HTMT), introduced by Fornell and Larcker [118] and Henseler et al. [119], respectively.

The Fornell-Larcker criterion establishes discriminant validity by comparing the square root of the Average Variance Extracted (AVE) for each latent variable with its correlations with other variables. If the square root of a construct’s AVE is greater than its highest correlation with any other construct, discriminant validity is confirmed. The data presented in Table 3 demonstrate that for all constructs, the square roots of their AVEs exceed their respective inter-construct correlations. This finding satisfies the Fornell-Larcker criterion, confirming that the constructs are adequately distinct from each other.

**Table 3:** Fornell and Larcker.

	AR	ED	EH	ES	ET	OIB	PL
AR	0.907						
ED	0.765	0.887					
EH	0.730	0.716	0.886				
ES	0.689	0.694	0.649	0.896			
ET	0.783	0.819	0.738	0.694	0.878		
OIB	0.758	0.673	0.637	0.667	0.701	0.882	
PL	0.850	0.757	0.699	0.645	0.771	0.704	0.893

*Note:* ET = Entertainment Experience, ED = Education Experience, ES = Escapist Experience, EH = Esthetic Experience, AR = Arousal, PL = Pleasure, OIB = Online Impulse Buying.

The Heterotrait-Monotrait (HTMT) ratio method evaluates discriminant validity by measuring the ratio of between-construct correlations (heterotrait) to within-construct correlations (monotrait). According to Henseler et al. [119] and Garson [120], discriminant validity is established when the HTMT value remains below 1. In this study, as shown in Table 4, all HTMT values meet this criterion, confirming that the constructs maintain discriminant validity. Although two HTMT values (ET-ED: 0.917 and AR-PL: 0.921) slightly exceed 0.9, this does not necessarily indicate a violation of discriminant validity. Moreover, in research fields where constructs are theoretically related but still distinct, such as psychology and social sciences, higher correlations are expected and do not automatically indicate poor discriminant validity [119]. While the conventional HTMT threshold is set below 0.85 or 0.9, some researchers (e.g., Voorhess et al. [121], Hair et al. [115], Garson [120]) argue that discriminant validity remains acceptable as long as HTMT values do not reach 1. Their reasoning is based on the premise that perfect collinearity occurs only when HTMT equals 1, implying that constructs become indistinguishable. Therefore, an HTMT value approaching but not reaching 1 suggests a strong relationship without necessarily compromising discriminant validity. Furthermore, the robustness of discriminant validity in this study is further supported by additional checks, including the HTMT bootstrap confidence intervals and the Fornell-Larcker criterion, both of which consistently confirm the distinctiveness of the constructs. Given this broader acceptance, the results affirm that the necessary criteria for discriminant validity are met, as none of the HTMT values reach or exceed 1.

**Table 4:** Heterotrait-monotrait ratio (HTMT).

	AR	ED	EH	ES	ET	OIB	PL
AR							
ED	0.849						
EH	0.794	0.801					
ES	0.744	0.756	0.710				
ET	0.856	0.917	0.815	0.762			
OIB	0.826	0.747	0.702	0.732	0.775		
PL	0.921	0.836	0.766	0.702	0.849	0.772	

*Note:* ET = Entertainment Experience, ED = Education Experience, ES = Escapist Experience, EH = Esthetic Experience, AR = Arousal, PL = Pleasure, OIB = Online Impulse Buying.

### 4.2. Structural model results

#### 4.2.1. Collinearity statistics.

This study utilized a self-response survey to collect data on both predictor and outcome variables. While this approach is practical, it can introduce common method bias, a concern highlighted by Hair et al. [115]. Common method bias arises when respondents provide similar answers due to overlapping content in the survey or a tendency to respond in socially desirable ways, leading to artificially inflated correlations among variables. This can create the appearance of relationships between constructs that are not genuinely related.

To address this issue, the study meticulously applied collinearity statistics, focusing on the Variance Inflation Factor (VIF), to assess the presence of multicollinearity among the independent latent variables. Multicollinearity can compromise the accuracy of the model by inflating standard errors and distorting parameter estimates. According to Hair et al. [115], a VIF score above 5 suggests a high likelihood of multicollinearity. However, as demonstrated in Table 5, all VIF values recorded are below this threshold, indicating no significant multicollinearity concerns among the latent variables. This result confirms that the issue of multicollinearity has been effectively addressed, thereby enhancing the model’s overall validity and reliability.

**Table 5:** Structural model assessment.

Hypothesis	Structural	Original Sample (O)	Standard Deviation	T Statistics	P Values	VIF	Results
H1	ET → AR	0.291	0.075	4.005	0.000	3.769	Accepted
H2	ET → PL	0.170	0.070	2.388	0.016	4.055	Accepted
H3	ED → AR	0.265	0.060	4.526	0.000	3.586	Accepted
H4	ED → PL	0.128	0.059	2.186	0.031	3.824	Accepted
H5	ES → AR	0.163	0.050	3.218	0.001	2.201	Accepted
H6	ES → PL	0.008	0.043	0.181	0.856	2.291	Rejected
H7	EH → AR	0.218	0.061	3.656	0.000	2.531	Accepted
H8	EH → PL	0.063	0.043	1.453	0.141	2.691	Rejected
H9	AR → PL	0.566	0.067	8.448	0.000	3.381	Accepted
H10	AR → OIB	0.576	0.069	8.151	0.000	3.598	Accepted
H11	PL → OIB	0.215	0.073	2.909	0.003	3.598	Accepted

*Note:* ET = Entertainment Experience, ED = Education Experience, ES = Escapist Experience, EH = Esthetic Experience, AR = Arousal, PL = Pleasure, OIB = Online Impulse Buying.

#### 4.2.2. Hypotheses assessments.

**Fig 2:**
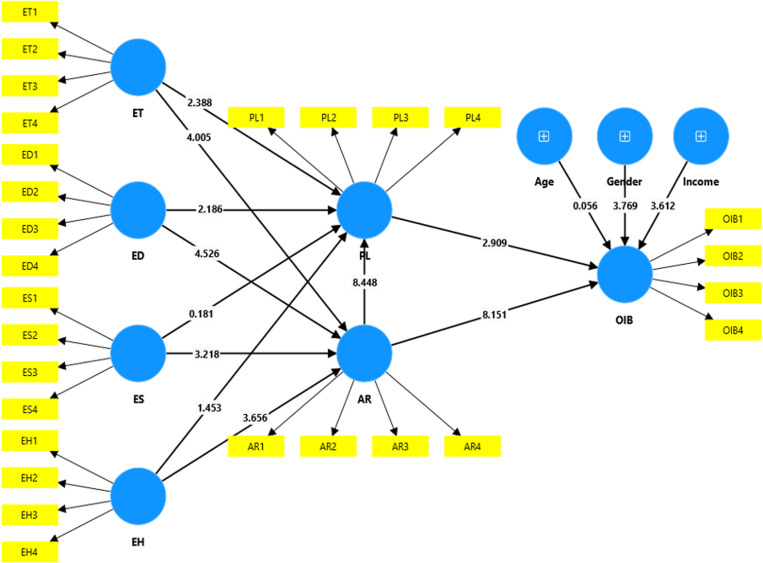
PLS-SEM Inner & Outer Model.

Table 5 and Fig 2 shows the results of the hypothesis testing. Firstly, the results show that entertainment experience (ET) significantly enhances both arousal (AR) and pleasure (PL), supporting H1 (O = 0.291, p = 0.000) and H2 (O = 0.170, p = 0.016). These findings suggest that an enhanced entertainment experience leads to higher levels of consumer arousal and pleasure. Similarly, education experience (ED) has a significant effect on both arousal (AR) and pleasure (PL), supporting H3 (O = 0.265, p = 0.000) and H4 (O = 0.128, p = 0.031). The results indicate that the positive effects of education experience consistently promote feelings of arousal and pleasure. Moreover, the analysis results support H5 (O = 0.163, p = 0.001), indicating a positive influence of escapist experience (ES) on arousal (AR), while rejecting H6 (O = 0.008, p = 0.856) which indicate insignificant effect of escapist experience (ES) on pleasure (PL). This implies that although escapist experience may arouse customers, they do not necessarily contribute to their pleasure. Similarly, esthetic experience (EH) positively affects arousal (AR), supporting H7 (O = 0.218, p = 0.000), but does not significantly impact pleasure (PL), leading to the rejection of H8 (O = 0.063, p = 0.141) This implies that while esthetic experience enhances arousal, it does not significantly motivate consumer pleasure. Furthermore, the results show that arousal (AR) has a significant positive impact on both pleasure (PL) and impulse buying behavior (OIB), supporting H9 (O = 0.566, p = 0.000) and H10 (O = 0.576, p = 0.000). These findings demonstrate that as arousal levels rise, so do consumers’ pleasure and their tendency towards impulse buying behavior. Notably, H10, with the highest O value of 0.576, highlights that arousal (AR) has the most significant effect on impulse buying behavior (OIB). Finally, the results support H11 (O = 0.215, p = 0.003), demonstrating that pleasure (PL) positively influences impulse buying behavior (OIB). The findings indicate that as consumers perceive a high level of pleasure when watching product introduction videos, their inclination towards making impulsive purchases also escalates.

#### 4.2.3. The effects of control variables.

Table 6 presents the results of impact of control variables on online impulse buying (OIB). The analysis reveals that age does not have a significant effect on OIB, as indicated by the rejection of hypothesis H12 (a) (O = -0.002, p = 0.955). In contrast, both gender and income significantly influence OIB, as supported by the acceptance of hypotheses H12 (b) (O = 0.108, p = 0.001) and H12 (c) (O = 0.105, p = 0.001). These findings suggest that gender and income directly affect online impulse buying, with variations in impulsive behavior observed across different gender and income groups. However, age does not play a significant role, indicating that consumers across different age groups tend to exhibit similar levels of impulse buying behavior.

**Table 6:** Control variables effects.

Hypothesis	Structural	Original Sample (O)	Standard Deviation	T Statistics	P Values	Results
H12 (a)	Age - > OIB	-0.002	0.029	0.056	0.955	Rejected
H13 (b)	Gender - > OIB	0.108	0.031	3.769	0.001	Accepted
H14 (c)	Income - > OIB	0.105	0.031	3.612	0.001	Accepted

*Note:* OIB = Online Impulse Buying.

#### 4.2.3. Evaluation of explanatory power (R square) and predictive power (Q square) of the research model.

The study evaluated the model’s explanatory and predictive power using R-squared and Q-squared indices, which play a crucial role in reflecting the model’s performance [122]. The R-squared index measures the percentage of variance in the dependent variable explained by the independent variables, while the Q-squared index assesses out-of-sample prediction accuracy. By prioritizing the adjusted R-squared, the study can achieve a more accurate estimation of explanatory power as it adjusts for the number of predictors.

Research results show that the highest adjusted R-squared value was observed for Pleasure (PL) at 0.755, indicating that the independent variables explain 75.5% of the variance in PL. Arousal (AR) had the second highest adjusted R-squared at 0.702, explaining 70.2% of its variance. Impulse buying behavior (OIB) had the lowest adjusted R-squared at 0.586, suggesting that the independent variables account for 58.6% of its variance. Hair et al. [123] noted that the R-squared value illustrates the explanatory power of the independent variables on a dependent variable. However, assessing the model’s predictive ability requires more than R-squared alone, as it may only partially capture characteristics beyond the study sample. To address this concern, Stone [124] and Geisser [125] introduced the Q-squared index to evaluate out-of-sample predictive power. The Q-squared value for OIB is 0.452, falling within the 0.25–0.5 range, indicating average predictive accuracy. Meanwhile, the Q-squared values for PL and AR are 0.596 and 0.574, respectively, suggesting high predictive accuracy for these models.

The findings presented in this section provide a thorough assessment of the measurement and structural models, affirming the research’s robustness. The analysis confirms the validity and reliability of the measurement model and highlights significant relationships among key variables in the structural model. Moreover, the structural model results illuminate the interrelationships among critical variables, uncovering intriguing influence patterns. The evaluation of explanatory and predictive power further strengthens the model’s credibility, showcasing its effectiveness in explaining variances and forecasting outcomes.

## 5. Discussion

This research examines the factors that affect online impulse buying behaviors, specifically targeting Generation Z consumers on the Shopee video platform. The study centers on the effects of internal stimuli including entertainment experience (ET), educational experience (ED), escapist experience (ES), and esthetic experience (EH) on impulsive buying, with emotional responses, namely arousal and pleasure acting as mediators. The study also reveals the role of demographic factors such as age, gender, and income serve as control variables.

The study’s results show that entertainment experiences significantly boost arousal (H1) and pleasure (H2), thereby enhancing consumer purchasing behavior. These results align with Jeong et al. [13], who found that positive entertainment experiences can elevate both pleasure and arousal among consumers. Similarly, Hosany and Witham [126] highlighted the significant increase in arousal that results from engaging entertainment experiences, underscoring the critical role of meeting consumer expectations in this context. However, the literature presents some conflicting views. For instance, Ryu and Park [127] found no significant impact of entertainment experiences on consumer pleasure, a discrepancy that may be attributed to their focus on an older demographic. This stands in contrast to the present study, which centers on Generation Z, a cohort characterized by its familiarity with on-demand entertainment. The unique attributes of Generation Z, who have grown up in a digital-first environment with immediate access to entertainment, likely account for the differing outcomes observed across studies. Research by Glapa et al. [128], Nagy et al. [47], and Spilková [129] supports this, suggesting that digital entertainment, a favored activity among Generation Z, enhances excitement and pleasure, subsequently driving impulsive purchasing behavior. In essence, the data indicate that entertainment experiences effectively boost arousal and pleasure, thereby fostering impulsive buying, especially on platforms like Shopee video, which are well-aligned with Generation Z’s preferences. This demographic’s inclination toward digital entertainment makes such platforms particularly potent in driving impulsive purchases. Additionally, cultural context plays a pivotal role in shaping consumer responses to entertainment experiences. For example, South Korea, the origin of the Hallyu wave, has set particularly high standards for entertainment quality [130]. These elevated expectations may diminish the pleasure derived from entertainment experiences among Korean consumers, potentially reducing the likelihood of impulsive buying. Conversely, Vietnam’s entertainment landscape is rapidly evolving, influenced by a diverse array of global trends [131]. This dynamic environment may foster a greater openness to novel forms of entertainment, creating a more conducive atmosphere for impulse buying behavior, particularly in the context of Vietnam’s rapid digitalization.

The study reveals that educational experiences significantly influence both arousal (H3) and pleasure (H4), aligning with the findings of Kastenholz et al. [16] and Goel et al. [132]. These researchers have shown that positive educational experiences enhance emotional responses by fostering consumer engagement, building confidence, and creating a sense of belonging. These elements collectively contribute to increased arousal and pleasure, suggesting that when consumers perceive educational content as valuable and engaging, it significantly boosts their emotional involvement in the shopping experience. In contrast, Singh et al. [133] report that educational experiences do not impact arousal and pleasure, indicating a perception among some consumers that shopping lacks educational value. This is further supported by Jeong et al. [13], who found that educational content focusing primarily on raw textual information can limit emotional responses, failing to engage consumers effectively. The variation in these findings may be attributed to cultural differences, particularly the influence of confucian values in Vietnam, which heightens sensitivity to educational content. This cultural context suggests that Vietnamese consumers are more likely to respond positively to educational experiences that resonate with their cultural expectations and values. Furthermore, the discrepancies in the impact of educational experiences may also be related to Generation Z’s expectations for continuous and engaging learning environments, as highlighted by Kahl [134]. This generation values discovery and reflection in their educational experiences, preferring content that actively involves them rather than passive consumption of information. Educational experiences that offer both active and passive engagement opportunities allow consumers to immerse themselves in useful information, thereby enhancing their emotional responses [135]. Additionally, educational experiences contribute to consumer convenience by providing essential product knowledge, enabling them to make informed decisions and efficiently complete their purchasing tasks. This, in turn, creates feelings of arousal and pleasure during shopping, as consumers feel more confident and satisfied with their choices [15].

The study’s results also indicate that escapist experiences (ES) significantly enhance arousal (AR) (H5) during purchases, which in turn promotes impulse buying behavior. This finding aligns with Güzel’s [136] research, which suggests that escapist activities, by offering a temporary diversion from reality, can heighten arousal and influence impulse buying. However, Hosany and Witham [126] did not find a significant effect of escapist experiences on arousal, which may be explained by generational differences. Generation Z, deeply engaged with the online world and digital social interactions, is particularly susceptible to the effects of escapism due to their increased levels of anxiety and depression [137,138].This heightened emotional sensitivity may amplify their arousal in response to escapist experiences. On the other hand, the study found that the impact of escapist experiences on pleasure (PL) (H6) was statistically insignificant. This contrasts with previous studies by Jeong et al. [13], Purwandari et al. [139], and Ryu and Park [127], which linked escapist experiences to increased emotional pleasure. Kuppens [83] suggests that high arousal might sometimes accompany negative emotions, which could explain why escapist arousal, while intense, may not always translate into pleasure. This indicates that while escapist experiences can trigger arousal, they might also reflect underlying anxiety, potentially diminishing overall pleasure.

Additionally, the research shows that esthetic experiences (ES) also enhance arousal (AR) (H7), with visual aesthetics playing a critical role in shaping consumer perceptions. Goel et al. [132] support this finding, emphasizing that attractive imagery in shopping apps encourages users to browse more and make purchases. Similar observations were made by Kastenholz et al. [16] and Liu and Zhang [49], who reported increased arousal linked to esthetic experiences. However, the influence of esthetic experience on pleasure (H8) was not statistically significant, diverging from findings by Jeong et al. [13] and Ryu and Park [127], who associated aesthetics with increased pleasure. Schindler et al. [140] suggest that the complexity of emotions triggered by esthetic experiences, sometimes involving negative feelings, might explain this variance. Further research by Wagner et al. [141] and Vuoskoski and Eerola [142] also indicates that adverse emotional states can negatively correlate with esthetic appreciation. Therefore, while esthetic experiences can boost arousal and promote impulsive purchases, they might under certain circumstances reduce pleasure. Therefore, platforms like Shopee video should customize esthetic content to align with individual customer preferences for optimal effectiveness.

Moreover, the research has uncovered a strong link between arousal (AR) and pleasure (PL) (H9), demonstrating that increased arousal significantly enhances pleasure, especially during impulsive online purchases. This finding is consistent with previous studies by Goel et al. [132] and Loureiro [143], which also identified that heightened arousal boosts emotional engagement, thereby increasing the perceived value and pleasure of a product or experience. These studies suggest that heightened arousal increases emotional engagement, which in turn amplifies the perceived value and pleasure of a product or experience, leading consumers to derive positive feelings from their purchase decisions. Yang et al. [49] and Miniero et al. [144] further explain that arousal can directly lead to pleasure, reinforcing the interconnectedness of these emotions. These observations are particularly pertinent in the context of online shopping, where emotional states can significantly impact purchasing behavior. When consumers experience arousal while shopping online, perhaps triggered by attractive experiences, this state can enhance the overall sense of pleasure. This pleasure can make the shopping experience more satisfying, potentially leading to impulsive online buying decisions as the immediate emotional gratification outweighs rational considerations.

Furthermore, the study underscores the critical roles of arousal (AR) (H10) and pleasure (PL) (H11) in driving online impulsive buying. Emotional stimuli, triggered by various experiences such as entertainment, educational, escapism, and esthetic, play a significant role in influencing consumers’ impulse to purchase. This finding is supported by the work of Lin and Lo et al. [145], who also found a strong link between arousal, pleasure, and impulsive online buying. Specifically, emotional surges can prompt spontaneous purchasing decisions, often without careful consideration, as the desire to maintain positive feelings outweighs more deliberate, logical thinking. Shen and Khalifa [146] corroborate this by noting that emotional stimulation frequently leads to impulsive purchases. Similarly, Ju and Ahn [147] observed that individuals experiencing pleasure are more prone to make impulsive purchases without much deliberation. Overall, the study reveals that arousal and pleasure, especially when connected to emotionally resonant content, be it entertainment, educational, esthetic, or escapist, which are key drivers of impulse buying behavior in online environments.

Importantly, the research found that gender and income significantly influence online impulse buying behavior (H12b, H12c), verifying their roles in controlling impulsive purchasing tendencies. These factors determine an individual’s likelihood of making spontaneous purchases online. The increasing diversity in gender roles and identities has a notable impact on consumer behavior, particularly in the context of impulsive buying. Studies consistently show that male and female consumers exhibit different levels of impulse buying behavior, influenced by various psychological and social factors. This observation is consistent with Lavuri’s [148] research, which suggests that gender is a crucial determinant of impulse buying behavior. Atulkar and Kesari [149] found that female consumers are generally more prone to impulsive buying than males, which can be attributed to factors like a higher fear of missing out (FOMO), stronger hedonic shopping motivations, and greater materialism, as noted by Chetioui & Bouzidi [150]. Additionally, men and women may differ in the types of products they are inclined to purchase impulsively, as observed by Pradhana and Sastiono [151]. Income levels also significantly affect online impulse buying. Individuals with higher incomes are more likely to engage in impulsive purchases due to their greater disposable income, which allows them to afford unplanned expenses without financial strain [107]. Conversely, those with lower incomes tend to be more cautious with their spending due to budget constraints. These findings align with previous studies by Jiang et al. [152] and Lavuri [148], which also noted that income influences impulsive shopping behaviors in different ways. Interestingly, age does not appear to significantly affect online impulse buying (H12a). Generation Z, in particular, shows a consistent pattern of impulse buying behavior, driven by their familiarity with digital platforms and a preference for instant gratification [98]. This suggests that Generation Z’s impulse buying behavior is influenced more by shared experiences and digital engagement rather than age differences.

Overall, this study emphasizes the importance of e-commerce platforms strategically designing content to harness these emotional responses, especially among digital natives like Generation Z. The findings highlight the need for a nuanced approach in content creation that aligns with consumer preferences and emotional triggers to enhance the effectiveness of online marketing strategies, ultimately driving consumer engagement and impulsive buying.

## 6. Implications

### 6.1. Theoretical implications

This study offers valuable insights into Generation Z’s online impulse buying behavior by exploring the key factors that influence these behaviors. While previous research has largely focused on the impact of product videos on sales and purchase intentions, it often neglected the emotional responses these videos evoke [153]. This study addresses this gap by investigating the complex relationship between internal factors, such as entertainment, educational, escapist, and esthetic experiences, and the emotional states of arousal and pleasure they trigger.

By integrating emotional responses as a mediating factor in impulsive purchasing within digital environments, this research extends the existing literature, drawing upon several well-established theoretical frameworks. Firstly, the study explores how internal stimuli on e-commerce platforms activate emotional states that subsequently drive impulsive buying decisions. This aligns with the Pleasure-Arousal-Dominance (PAD) model of Mehrabian and Russell [84], which categorizes human responses to stimuli into pleasure and arousal during decision-making processes. The findings underscore the critical role of arousal and pleasure in shaping impulsive online buying behavior. Secondly, the research supports the Experience Economy theory (4Es) proposed by Pine and Gilmore [35], demonstrating the significant impact of experiential factors such as entertainment, education, escapism, and esthetics on emotional responses. These factors, in turn, enhance consumers’ impulsive purchase intentions. The study also highlights the importance of creating engaging and positive experiences in online shopping environments, where interactive entertainment and promotional programs serve as key drivers of impulse buying behavior. Furthermore, this research aligns with Flow theory by Csikszentmihalyi’s [41], illustrating how deep immersion in online shopping experiences facilitates smoother actions and enhances positive emotions. This immersion has important implications for consumer behavior, optimizing the shopping experience and fosters engagement. As consumers become more absorbed in the shopping experience, they are more receptive to impulse buying triggers. This heightened state of focus and enjoyment can amplify the impact of these triggers, prompting quicker decision-making and reducing hesitation in completing a purchase. Additionally, the study validates Arnold and Reynolds’ [154] theory of hedonic shopping motivations, emphasizing the importance of emotional and pleasure-driven reasons in driving impulsive purchases across various e-commerce platforms. According to this theory, consumers often shop not just for functional reasons but to fulfill emotional needs, such as the desire for excitement, escape, or self-reward. These hedonic motivations lead shoppers to seek pleasure and enjoyment in the shopping experience itself, making them more likely to make impulsive purchases. Moreover, the findings support Fredrickson’s [155] broaden-and-build theory of positive emotions, demonstrating how positive stimuli, such as appealing escapist and esthetic experiences, enhancing cognitive and emotional responses. In the context of online shopping, esthetically appealing and immersive features like vibrant visuals, engaging narratives, and a seamless user interface evoke positive emotions that heighten the shopping experience. These elements can make the online environment feel enjoyable and even escapist, drawing consumers into a state where they feel relaxed, entertained, and receptive. This emotional uplift broadens cognitive engagement, allowing consumers to explore products more freely and imaginatively, consider a wider variety of items, and ultimately deepen their connection to the shopping experience. Finally, the study affirms aspects of Thaler and Sunstein’s [156] Nudge theory, suggesting that subtle, well-designed experiences in the shopping environment can effectively influence consumers’ purchasing decisions. Nudge theory posits that small cues or “nudges”, such as gentle prompts or suggestions, can influence decision-making without restricting choices. In e-commerce, these nudges might include elements such as product recommendations, or visually highlighted items, which subtly guide consumers toward certain products or actions while maintaining a sense of freedom in their decision-making. This highlights the diverse and complex nature of online impulse buying behavior.

### 6.2. Managerial implications

This study provides critical insights into the factors that drive impulsive purchasing behaviors among Generation Z, particularly their strong responsiveness to dynamic and engaging content on digital platforms like Shopee. As digital platforms increasingly shape consumer behaviors, it is essential for companies to conduct thorough research into the preferences and behavioral patterns of this demographic [157]. Strategic identification and targeting of the right audience, combined with leveraging impulsive buying trends, are key to achieving marketing success in today’s competitive landscape.

To effectively capitalize on these trends, companies must develop and implement online marketing strategies that align with their brand values. This approach particularly appeals to value-driven demographics like Generation Z, fostering stronger connections and loyalty. This alignment fosters trust and loyalty, as customers are more likely to engage with brands whose messaging feels genuine and consistent. Additionally, a values-based approach enables companies to stand out in a crowded market, attracting and retaining customer attention through meaningful connections rather than purely transactional interactions. Creating entertaining and interactive content, such as quizzes and games embedded within promotional videos, can significantly enhance emotional engagement. A quiz that helps users discover products tailored to their tastes or a game that offers discounts as rewards provides a sense of accomplishment and connection with the brand. Which can foster trust in the brand because it positions the brand as a source of enjoyment and valuable information, rather than solely a promoter of products. This way fosters strong brand relationships and builds trust in the shared information, as supported by Hollebeek et al. [158]. Marketers should also promote hashtag campaigns that encourage consumers to share their product experiences, creating deep emotional connections and memorable experiences that boost customer engagement. Building emotional connections through personalized and escapist experiences can further maximize users’ emotional responses. Tailoring experiences to individual preferences and providing an element of escapism, brands make users feel more connected and captivated, encouraging spontaneous purchasing decisions. Tapping into consumers’ emotions, making the shopping journey not only satisfying but also memorable, thereby driving impulse buying behavior. Given that Generation Z primarily interacts with content on mobile devices, marketers must prioritize a mobile-first design approach. Ensuring a balanced layout between content and images within a frame enhances user experience, making it easier for consumers to engage with content and make purchases. This approach not only attracts consumers to the brand but also drives impulse buying behavior.

Demographic factors such as age, gender, and income should be carefully considered when designing marketing strategies. The study reveals that age does not directly influence online impulse buying behavior among Generation Z; instead, this demographic is highly responsive to content within the first five seconds of a video. To effectively engage Generation Z, marketers should focus on creating concise, impactful videos that incorporate humor and surprising elements to capture attention immediately. Such an approach aligns with Generation Z’s preference for fast-paced, engaging content, thereby enhancing the potential for impulse purchases. By employing this strategy, brands can effectively stimulate impulse buying behaviors within this demographic. Gender differences also play a significant role in online impulse buying behavior. For women, high-quality videos with engaging visuals and emotional messages are particularly effective. Videos that incorporate brand stories, positive emotions, and real-life situations resonate well with this demographic. Businesses should consider creating videos that subtly integrate customer testimonials and product success stories to appeal to female consumers. Conversely, men are often drawn to videos that emphasize product features, performance, and innovation. Businesses should highlight the technical aspects and practical benefits of their products, while also providing detailed product reviews and comparison videos. This approach can help male consumers better understand the product and its value, thereby promoting online impulse buying behavior within this audience. Income levels also significantly influence online impulse buying behavior. High-income earners tend to prioritize exclusive benefits and special product information. To cater to this segment, businesses should create exclusive customer loyalty programs, offer special discounts, and provide priority membership options for ordering new collections. On the other hand, low-income earners are more cautious with their spending and tend to prioritize economic considerations. Integrating educational video content that explains the benefits and uses of a product can help these consumers gain a deeper understanding and trust, ultimately increasing their engagement and impulse buying intentions.

In summary, customizing content to match the interests and needs of Generation Z is essential for maximizing its relevance and impact. This approach not only creates a unique brand identity that stands out from competitors but also improves customer satisfaction, increases perceived product value, fosters customer loyalty, and enhances the overall effectiveness of digital marketing strategies in the ever-evolving e-commerce landscape.

## 7. Limitations and recommendations

Although this study has made contributions to both theoretical and practical aspects, it is essential to acknowledge its limitations. Research mainly focuses on internal experiential factors but ignores external factors that influence online impulsive shopping behavior. Future studies should incorporate and expand the analysis of internal and external factors influencing online impulsive buying across multiple e-commerce platforms. This approach may enhance the generalizability of the findings and provide a more comprehensive understanding of diverse online shopping environments.

Furthermore, it is essential to note that the study’s sample is predominantly from Generation Z and limited in diversity due to geographical, temporal, and funding constraints. Future research should include a more diverse demographic profile, spanning different geographic locations and age groups, to ensure higher objectivity and comprehensiveness. This expansion would be instrumental in comparing behaviors across different demographic segments, thereby enhancing the credibility of the findings.

The research could significantly benefit from exploring potential moderating variables such as personality traits, or situational variables. This could lead to a more nuanced understanding of the dynamics between the studied factors and impulse buying behavior.

To mitigate potential response biases, incorporating additional data sources like observational data, online behavior tracking, or qualitative interviews could complement the survey data. Expanding the research across different cultural contexts would provide valuable insights into how consumer behavior and preferences vary with cultural factors. These efforts lay a robust foundation for marketers to engage effectively with diverse consumer segments, ultimately enhancing the applicability and depth of future research.

## 8. Conclusion

This research delves into the dynamics of online impulse buying behavior among Generation Z on the Shopee video platform, proposing a model to examine how internal factors, including entertainment (ET), educational (ED), escapist (ES), and esthetic (EH) experiences influence impulsive buying through emotional responses. Demographic factors will serve as control variables. The analysis employed the SmartPLS statistical tool, a robust method for structural equation modeling that elucidates complex variable relationships.

Key findings illustrate that entertainment and educational experiences significantly affect arousal and pleasure, highlighting their role in fostering impulsive buying. Entertainment experiences elevate pleasure and arousal through engaging and humorous product presentations, creating a positive shopping ambiance that heightens impulsive purchasing tendencies. Similarly, educational experiences enrich product knowledge, enhancing customer pleasure, arousal, and purchasing confidence.

While escapist experiences boost arousal, their effect on pleasure is not statistically significant, indicating varied impacts on consumer behavior. These experiences often introduce novel product explorations that increase arousal due to new and appealing product features, subsequently enhancing impulsive buying. Esthetic experiences increase arousal through visually appealing presentations and high-quality imagery, captivating viewers. While these experiences generally enhance arousal, they do not significantly impact pleasure. In some instances, individuals in a negative mood may find that esthetic experiences exacerbate feelings of displeasure.

The study underscores that both arousal and pleasure crucially steer impulsive buying, with arousal having a more pronounced effect and a more substantial influence on online impulse buying compared to pleasure. To extend the findings, this study also explored the significant impact of demographic variables such as age, gender, and income on online impulse buying behavior. The findings revealed that gender and income positively influence online impulse buying, while age was statistically insignificant. Female consumers are more frequent shoppers and are more emotionally driven, making impulse purchases than any other gender. They are drawn to beautiful, unique products and are willing to spend money on experiences, driven by the desire to satisfy immediate needs and keep up with trends. Notably, high-income earners, less burdened by financial worries, comfortably indulge in impulse purchases without overthinking the financial consequences. In contrast, low-income earners are less confident about their financial capabilities, leading to careful consideration of each purchase. This highlights the important role of emotional responses and demographic variables in driving impulse buying decisions on digital platforms.

These insights are invaluable for marketers, researchers, and e-commerce professionals, offering strategies to optimize emotional responses and promote impulsive buying on digital platforms. Moreover, the research stresses the importance of balancing experiential elements to maximize arousal and pleasure among Generation Z consumers, thus enriching the online shopping experience and impulsivity on platforms like Shopee video. This investigation significantly contributes to understanding online consumer behavior, enabling the development of potent marketing strategies tailored for e-commerce environments.

## Supporting information

S1 File Survey questionnaire. (PDF)

S2 File Dataset used in analysis. (XLSX)
