# Supplementary material for: Analyzing the role of customers’ experiences and emotional responses in shaping Generation Z’s impulse buying behavior on Shopee video platform
Source: PLoS One. 2025 May 5;20(5):e0322866. doi: 10.1371/journal.pone.0322866 (PMC12052140; doi:10.1371/journal.pone.0322866)
Supplement: S1 File — (PDF) [file pone.0322866.s001.pdf]

QUESTIONNAIRE

Analyzing the role of customers' experiences and emotional responses in shaping Generation Z's impulse buying behavior on Shopee video platform

This study investigates the impact of product presentation videos on Shopee on customers' online impulse buying behavior. With the rapid rise of e-commerce, especially in Southeast Asia, short-form video content has become a key driver of this growth. Our research seeks to bridge the existing knowledge gap by exploring how internal factors, including entertainment, educational, escapist, and esthetic experiences, influence consumers' arousal and pleasure, which in turn mediate their impulse buying behavior. Additionally, the study examines how demographic factors play a controlling role. The findings will provide valuable insights for developing marketing strategies on Shopee that effectively target customers' emotions and influence their online shopping decisions.

We sincerely appreciate your assistance in completing this survey. Participation is entirely voluntary and anonymous. We assure that all your information will be kept confidential and used solely for research purposes.

Do you agree to the use of the information provided for research purposes?

- YES
- NO

Have you ever made a random purchase on Shopee video?

- YES
- NO

I. Personal Information

1. Gender:

- Male (1)
- Female (2)

2. What is your year of birth?

- From 1997 - 2001 (1)
- From 2002 - 2006 (2)

3. What is your educational level?

- High school (1)
- College (2)
- University (3)
- Postgraduate (4)
- Other:______ (5)

4. What is your occupation?

- Pupils/students (1)
- Teachers (2)
- Business (3)
- Office staff (4)
- Freelancers (5)
- Other:______ (6)

5. How much is your monthly income? (Including allowance from family, overtime pay, other sources, etc.)

- Under 5 million (1)
- From 5 - under 10 million (2)
- From 10 - under 15 million (3)
- From 15 - under 20 million (4)
- Over 20 million (5)

II. Online Buying Activities

1. What items do you often shop on Shopee video?

- Cosmetics (1)
- Household appliances (2)
- Electronic equipment (3)
- Fashion items (clothes, shoes, accessories, etc.) (4)
- Other:______ (5)

2. How often do you make random purchases on Shopee video over a month?

- Less than 3 times (1)
- From 3 to 5 times (2)
- More than 5 times (3)

III. Factors affecting impulsive buying behavior on Shopee video

Please check () and honestly assess yourself based on your actual behavior given the statements, using the following scales:

1 - Strongly Disagree to 5 - Strongly Agree

**No.**	**Criteria**	**Level of consent**				
		**Strongly Disagree → Strongly Agree**				
**I. Entertainment experience (Oh, Fiore and Jeoung, 2007)**						
**1**	ET1: “The way products were presented on Shopee video was amusing to me.”	1	2	3	4	5
**2**	ET2: “The way products were presented on Shopee video was very entertaining.”	1	2	3	4	5

**3**	ET3: "I really enjoyed looking at the new product presentations on Shopee video."	1	2	3	4	5
**4**	ET4: "I feel that it is pleasant to use Shopee video for shopping."	1	2	3	4	5
**II.**	**Educational experience (Oh, Fiore and Jeoung, 2007)**					
**1**	ED1: "The product presentations created a shopping experience that was educational to me."	1	2	3	4	5
**2**	ED2: "Browsing product presentations on Shopee video stimulated my curiosity to learn new things about products."	1	2	3	4	5
**3**	ED3: "The product presentations on Shopee video have made me more knowledgeable about products."	1	2	3	4	5
**4**	ED4: "I learned about products while browsing the product presentations on Shopee video."	1	2	3	4	5
**III.**	**Escapist experience (Oh, Fiore and Jeoung, 2007)**					
**1**	ES1: "When looking at the product presentations on Shopee video, I felt I was in a different world."	1	2	3	4	5
**2**	ES2: "I felt like I was a different person while looking at the product presentations on Shopee video."	1	2	3	4	5
**3**	ES3: "I totally forgot about my daily routine while looking at the product presentations on Shopee video."	1	2	3	4	5
**4**	ES4: "While looking at the product presentations on Shopee video, I completely escaped from reality."	1	2	3	4	5
**IV.**	**Esthetic experience (Oh, Fiore and Jeoung, 2007)**					
**1**	EH1: "The product presentations of Shopee video provided pleasure to my senses."	1	2	3	4	5
**2**	EH2: "Shopee video's product presentations were very attractive."	1	2	3	4	5
**3**	EH3: "Shopee video product presentations really showed attention to design detail."	1	2	3	4	5
**4**	EH4: "Just looking at the product presentations on Shopee video, it was very pleasant."	1	2	3	4	5
**V.**	**Arousal (Hsich *et al.*, 2014; Huang *et al.*, 2017)**					

**1**	AR1: “When I was shopping on Shopee video, I felt excited.”	1	2	3	4	5
**2**	AR2: “When I was shopping on Shopee video, I felt active.”	1	2	3	4	5
**3**	AR3: “When I was shopping on Shopee video, I felt aroused.”	1	2	3	4	5
**4**	AR4: “When I was shopping on Shopee video, I felt stimulated.”	1	2	3	4	5
**VI. Pleasure (Hsich *et al.*, 2014; Kim *et al.*, 2016; Huang *et al.*, 2017)**						
**1**	PL1: “When I was shopping on Shopee video, I felt happy.”	1	2	3	4	5
**2**	PL2: “When I was shopping on Shopee video, I felt pleased.”	1	2	3	4	5
**3**	PL3: “When I was shopping on Shopee video, I felt satisfied.”	1	2	3	4	5
**4**	PL4: “When I was shopping on Shopee video, I felt hopeful.”	1	2	3	4	5
**VII. Online Impulse Buying (Rook and Fisher, 1995; Mehrabian and Russelk, 1974)**						
**1**	OIB1: “I usually buy products on Shopee video spontaneously.”	1	2	3	4	5
**2**	OIB2: “The products I bought on Shopee video are mostly unplanned.”	1	2	3	4	5
**3**	OIB3: “I bought a product on Shopee video that I did not initially want to buy.”	1	2	3	4	5
**4**	OIB4: “I sometimes cannot suppress the feeling of wanting to buy something online.”	1	2	3	4	5

Thank you for your support.

Research Team.
